# Generation of Asynaptic Mutants in Potato by Disrupting *StDMC1* Gene Using RNA Interference Approach

**DOI:** 10.3390/life13010174

**Published:** 2023-01-06

**Authors:** Ashwani Kumar, Sundaresha Siddappa, Vinay Bhardwaj, Dalamu, Baljeet Singh, Neha Sharma, Bhawna Dipta, Vinod Kumar, Umesh Goutam, Salej Sood

**Affiliations:** 1 ICAR-Central Potato Research Institute, Shimla 171001, India; 2 Department of Bioengineering and Biosciences, Lovely Professional University, Phagwara 144411, India

**Keywords:** Asynapsis, cross-over, *StDMC1*, meiosis, recombination, RNAi

## Abstract

Fixing the genomic composition and multiplication through true potato seed (TPS) is an important challenge in autotetraploid potato. Disrupted meiotic cDNA (DMC1) is a meiotic gene that plays a central role in DNA recombination through crossing over in meiosis. Using the *Arabidopsis* DMC1 (*AtDMC1*) gene sequence, we retrieved *Solanum tuberosum* DMC1(*StDMC1*) from the diploid potato genome, and subsequently, sense and antisense regions of the *StDMC1* gene were amplified in potato cv. Kufri Jyoti. The sense and antisense fragments were confirmed by Sanger-sequencing and cloned in the pRI101 vector. *Agrobacterium*-mediated transformation of the RNAi construct resulted in 44% transformation efficiency, and a total of 137 mutant lines were obtained. These mutant lines were further validated through pollen viability testing, and selected lines were used for gene expression analysis. The acetocarmine-based pollen staining showed reduced pollen viability ranging from 14 to 21% in four DMC1 mutant lines (DMC4-37, DMC4-41, DMC6-20, and DMC6-21), as compared to the Kufri Jyoti control plants, which on average exhibited 78% pollen viability. The phenotypic data was supported by the reduced expression of the *StDMC1* gene in these four mutant lines compared to the control Kufri Jyoti. The results confirmed the generation of *StDMC1* knockdown lines. This is the first report of *StDMC1* mutant line generation in tetraploid potatoes and will be a step forward in generating non-recombinant mutants through sexual reproduction in potatoes.

## 1. Introduction

The potato (*Solanum tuberosum* L.) is the world’s third most important food crop after wheat and rice [1] and is grown in all major countries [2]. Most of the cultivated potato varieties are autopolyploid and extremely heterozygous [3]. In potato breeding, heterozygous varieties are crossed with donor heterozygous genotypes to pass on desirable features, and the desired genotypes are chosen and released as new varieties in the F1 generation [4]. The hybrids cannot be recreated in the form of true potato seed (TPS) by crossing the same parental lines since each TPS in the F1 generation is genetically unique in potato [4]. Potato varieties are maintained in vitro and propagated clonally [5]. This is a significant distinction between potato breeding and that of key food crops such as rice, wheat, and maize, where homozygosity is first achieved before new varieties are released as open-pollinated homozygous lines or hybrid varieties. A constant influx of new genes and allelic diversity into the *S. tuberosum* gene pool is required to improve potato genetics for diverse economic traits. However, the introgression of new genes changes the genetic background of a genotype or a variety in potatoes [6], which has also been observed in other vegetables and crop wild relatives (CWRs) [7].

Potato seed production currently faces considerable challenges, such as low multiplication rates, high storage and transportation costs, virus carry-over, and performance reductions over generations [8]. Heterozygous genotypes cannot be sexually reproduced to form botanical seeds or TPS in light of the fact that parental chromosomes will recombine to produce different combinations and the same genotype cannot be reconstituted [9]. The favorable allele combinations are lost in the sexual cycle due to the segregation of traits. This is the reason that potatoes are reproduced clonally to maintain the favorable allelic combinations of genotypes/varieties [10]. Although apomixes is considered a solution to fix heterozygotes, it has not been applied in practical plant breeding in any crop [11]. Thus, fixing the genomic composition and multiplication through TPS is an important challenge in potato breeding [12]. An alternate method is the suppression of recombination and identification of asynaptic mutant lines, which could possibly recreate the parental type gametes and true-to-type botanical seeds similar in genetic constitution to starting clone/hybrid. The development of haploids and doubled haploids from asynaptic gametes is further required to create a set of parental lines, which, upon selection and hybridization, will recreate the original cultivar.

Homologous recombination (HR) is required for preserving genomic integrity across all domains of life, and it is also required for the repair of planned double-strand breaks (DSBs) during meiosis [13]. Meiotic HR results in chromosome segregation because of crossovers, while the purpose of mitotic HR is to repair DNA damage without crossovers. In mitotic cells, crossovers are less desirable since they can result in loss of homozygosity and other chromosomal rearrangements [14]. Crossovers, on the other hand, are preferred during meiosis because they are required for chromosome segregation and serve to increase genetic variation among offspring. Meiosis is an important step in the sexual reproduction cycle. It is an atomic division in which the number of chromosomes is reduced to half, which is essential for the arrangement of haploid cells in living organisms that reproduce sexually [15].

Meiotic recombination protein disrupted meiotic cDNA (DMC1) is a homologue of the bacterial strand trade protein RecA. DMC1 assumes the focal job in homologous recombination in meiosis by amassing at the destination. The protein encoded by this gene is basic for meiotic HR. Hereditary recombination in meiosis assumes a significant role in creating an assorted variety of hereditary data and encourages the reductional isolation of chromosomes that must happen in the development of gametes during sexual multiplication.

Meiotic recombination requires two types of RecA-like proteins, DMC1 and RAD51, that play an important role in HR. RecA protein is essential for the repair of DSBs, while both Rad51 and DMC1 are essential to generate a crossover between homologous chromosomes, which confirms the segregation of the chromosomes at meiotic division I [16]. The first eukaryotic RecA homologues, RAD51 and DMC1, were reported in budding yeast [17]. RAD51 expresses during mitosis and meiosis, whereas DMC1 expresses only during meiosis [18]. DMC1 disruption results in defects in reciprocal recombination, inability to form synaptonemal complexes, accumulation of DSBs, and abnormal chromosome synapses, showing D-loop formation and strand exchange activities [19]. The plant DMC1 orthologues were reported in *Lilium longiflorum* [20], *Arabidopsis thaliana* [21,22], *Oryza sativa* [23,24,25,26], and *Hordeum vulgare* [27]. The concept of deficient mutants in the meiotic recombination process arrests the meiotic recombination, crossing over, and pairing of chromosomes. This is the first step of reverse breeding to generate asynaptic mutants through which the parental-type gametes can be recovered in the progenies to fix the genomic composition and multiplication through TPS. Keeping this in mind, the present study aimed to silence the *StDMC1* gene in the Kufri Jyoti variety of potato to generate asynaptic mutants.

## 2. Materials and Methods

### 2.1. Plant Materials

The study was conducted using a popular tetraploid Indian potato variety, “Kufri Jyoti”. Kufri Jyoti is an old potato variety (1968) developed by the ICAR-Central Potato Research Institute, Shimla, Himachal Pradesh, India, through clonal selection from a cross 3069d(4)/2814a(1). The variety has wide adaptability and is still popular among stakeholders. The virus-free in vitro tubes of Kufri Jyoti were obtained from the germplasm repository, Division of Crop Improvement and Seed Technology, ICAR-Central Potato Research Institute (CPRI), Shimla, Himachal Pradesh, India. The cultures were further multiplied in vitro on Murashige and Skoog media containing sucrose 30 g/L, calcium pantothenate 2 mL/L and gelrite 3 g/L). The pH of the media was adjusted to 5.7. A sufficient number of plants were generated for planting in a glass house. After 28 days of planting, leaf samples were collected for RNA isolation, and at the time of flowering, anthers and carpels were also collected for RNA isolation.

### 2.2. Identification of StDMC1 CDS, Sense and Anti-Sense Region

The *Arabidopsis thaliana AtDMC1* coding DNA sequence (CDS) was retrieved from the Arabidopsis Information Resource (TAIR) database. The potato *StDMC1* region was obtained by using the *AtDMC1* CDS as a query in the Potato Genome Sequencing Consortium (PGSC) database using the BLAST tool. The *StDMC1* CDS sequence was used to select the siRNA by using the bioinformatics tool siRNA-scan (http://bioinfo2.noble.org/RNAiScan.htm, accessed on 17 January 2021). Based on the CDS region, sense and antisense fragments were selected for RNAi construct development. The BLAST tool was used to ensure that there was no off-target silencing. The primers were designed using FastPCR version 4.0 [28] (Table 1).

### 2.3. Sense and Anti-Sense Amplification

**Figure 1:**
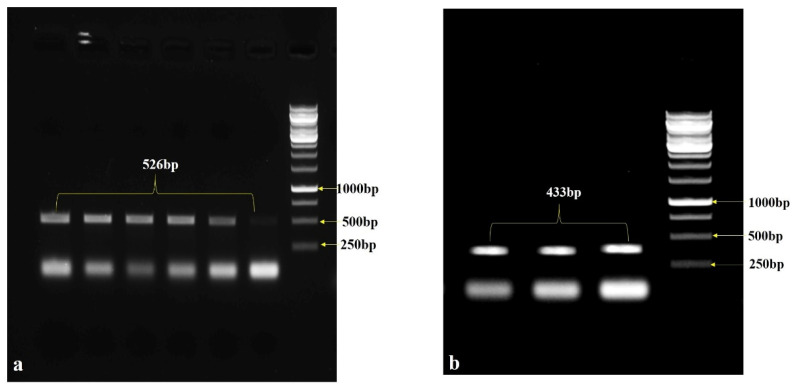
(**a**) PCR amplification of the *StDMC1* gene of a sense fragment with *StDMC* sense primers flanked with restriction sites *Sal* 1 and *Kpn* 1, resulting in a product size of 526 bp. (**b**) Amplification of an antisense gene fragment with *StDMC1* antisense primers flanked with restriction sites *Bam* HI and *Kpn* 1, resulting in a product size of 433 bp.

The RNA isolation was performed by a NucleoSpin Macherey-Nagel RNA Plant kit (GmbH & Co. KG, Germany). The cDNA was synthesized using a High-Capacity cDNA kit by Applied Biosystems^TM^ (Waltham, MA, USA). The sense and anti-sense regions were amplified using the reaction: 1 µL cDNA, 15 µL EmeraldAmp PCR master mix (Takara™, Kusatsu, Japan), 1 µL of each forward and reverse primer, and 2 µL of RNase-free water to make a final reaction volume of 20 µL. The PCR conditions were as follows: 4 min at 95 °C followed by 35 cycles of 45 s at 94 °C, 45 s at 54 °C and 1 min at 72 °C, and terminated with one cycle of 5 min at 72 °C (Figure 1a). The same PCR conditions were followed for the antisense region except for the annealing temperature (52 °C). The amplified fragments were resolved on a 1.5% agarose gel (Figure 1b), and the eluted PCR products were sequenced using the BigDye v3.1 reaction kit, Applied Biosystems^TM^ (USA) and analyzed on the ABI 3500 Genetic Analyzer, Applied Biosystems^TM^ (USA).

### 2.4. Development of siRNA Construct

The *StDMC1* sense fragment (526 bp) was amplified with gene-specific primers flanked with restriction sites for *Sal* 1 and *Kpn* 1, and the antisense gene fragment (433 bp) with restriction sites for *Bam* H1 and *Kpn* 1, respectively. These amplified PCR fragments were eluted from agarose gel and ligated in the pTZ57R/T TA cloning vector. The gene constructs pTZ57R/T::Sense *StDMC1* and pTZ57R/T::antisense were first cloned in *Escherichia coli* strain DH5α on a Luria Bertani (LB) agar plate containing Ampicillin (100 µg/mL). The colonies were confirmed via colony PCR as well as by restriction digestion. Later, these were subcloned into the binary vector pRI101AN. The sense plasmid and vector were digested with *Sal* 1 and *Kpn* 1 restriction enzymes for ligation. The ligated product was transformed using the heat shock method and maintained in *E. coli* strain DH5α selective media containing Kanamycin (50 µg/mL). The colonies obtained were confirmed through colony PCR using 35S forward and gene-specific reverse primers.

Similarly, the antisense fragment was amplified using antisense primers flanked with restriction sites for *Bam* H1 and *Kpn* 1 and subjected to restriction digestion for cloning in the *StDMC1* sense::pRI101AN vector backbone. The ligated products were maintained in *E. coli* strain DH5α. The confirmation of colonies obtained on selective LB agar plates containing Kanamycin was performed through colony PCR using 35S forward and NOST reverse primers. The whole cassette (*StDMC1*-sense::*StDMC1*-antisense::pRI101AN) was reconfirmed through restriction digestion using *Sal* 1 and *Bam* H1 enzymes.

### 2.5. Transformation of RNAi Construct in Agrobacterium Strain GV3101

The positive RNAi clone (*StDMC1*-sense::*StDMC1*-antisense::pRI101AN) was transformed into *Agrobacterium* strain GV3101 using the freeze-thaw method. *Agrobacterium* strain GV3101 carrying the RNAi gene cassette was grown in LB broth containing antibiotics (50 μg/mL of Kanamycin and 15 µg/mL of Rifampicin) in an incubator shaker at 28 °C for 48 h. From this culture broth, the plasmid was isolated using a Macherey-Nagel Nucleospin plasmid isolation kit (Germany). The transformed colonies were confirmed through restriction digestion using *Bam* H1 and *Hind* III enzymes.

### 2.6. Agrobacterium-Mediated Plant Transformation

The internodal stem cuttings of in vitro tissue-cultured plants were used as explant for genetic transformation. *Agrobacterium* culture harboring the *StDMC1* RNAi construct grown at 28 °C with an OD of 0.6, was used for transformation. For co-cultivation, the internodal cuttings were placed on pre-selective media containing basal MS medium for 48 h. Co-cultivation was performed with the *Agrobacterium* culture suspension obtained after centrifugation and resuspension in sterile MS liquid media. Acetosyringone (1 mg/mL) was added to the cell suspension containing cuttings to enhance virulence. The treated cuttings were dried using sterile tissue papers and then transferred to regeneration media containing Indoleacetic acid (IAA) (1 mg/mL), Naphthalene acetic acid (NAA) (0.01 mg/L), Gibberellic acid (GA3) (3 mg/mL), Cefotaxime (100 mg/mL), Kanamycin (50 µg/mL), and Carbenicillin (100 mg/L). The inoculated plates were kept at 20 ± 2 °C under fluorescent light at 100 μmol m^−2^ s^−1^ with 16 h of light and 8 h of dark until putative shoots emerged. These shoots were transferred into tubes containing MS media, Kanamycin (50 µg/mL), and IAA (300 µg/mL) for root initiation. The plantlets that survived were transplanted into the soil and grown under controlled conditions. Transformation efficiency was calculated using the following formula:

$$\mathrm{Transformation}\;\mathrm{efficiency}\;\left(\%\right)=\frac{\mathrm{Number}\;\mathrm{of}\;\mathrm{positive}\;\mathrm{transformants}}{\mathrm{Total}\;\mathrm{number}\;\mathrm{of}\;\mathrm{regenerated}\;\mathrm{plantlets}}\times 100$$

### 2.7. Screening of Putative Transformants

DNA was isolated from the leaves of transformed plants using a QIAGEN DNeasy kit as per the manufacturer’s instructions (North American manufacturing in Germantown, MD, USA). NPTII expression was tested by using NPTII forward and reverse primers. The PCR reaction contained 10 µL of Emerald PCR master mix (Takara™, Japan), 1 µL of each primer at a concentration of 1 μM, and 1 μL DNA templates in a reaction volume of 20 μL. PCR program was followed as 4 min at 94 °C; followed by 30 cycles 40 s at 94 °C, 50 s at 54 °C, 1 min at 72 °C, and 6 min at 72 °C. The transformed lines harboring the NPTII backbone were confirmed with an NPTII-specific primer on 1.3% agarose gel (Table 1).

### 2.8. Pollen Viability Analysis

The transformed lines as well as control plants were grown under controlled conditions. The flower buds were collected from transformed lines for pollen viability analysis. The pollen viability was tested using a 2% acetocarmine stain. An EVOS XL Core Imaging System (Invitrogen^TM^, Waltham, MA, USA) microscope was used to examine the pollen viability. Five frames of each slide were examined to count the viable and non-viable pollen grains. Viable pollen grains retained the stain, whereas the non-viable pollen grains were unable to retain the stain. The percent viability was calculated by dividing the amount of stained pollen grains per field of view by the total number of pollen grains in that view.

### 2.9. Quantitative Real-Time PCR (qRT-PCR)

After the confirmation of pollen viability, non-viable lines along with Kufri Jyoti controls were used for quantitative real-time PCR (qRT-PCR). The qRT-PCR was performed in triplicates in reference to the housekeeping gene elf (elongation factor). The qRT-PCR reaction mixture contained 10 μL of SYBER GREEN^®^ (Applied Biosystem^TM^, USA), 1 μL cDNA, 1 μL of each forward and reverse primer, and the final volume was adjusted by adding 2 μL dH_2_O (Table 2). Using the StepOnePlus^TM^ Real-Time PCR System (Applied Biosystems^TM^, USA), the expression of each gene, in comparison to the average Ct values of the housekeeping gene, was determined and analyzed. The 2^−ΔΔCt^ technique was used to quantitatively measure the relative changes in gene transcript levels [29].

## 3. Results

### 3.1. Characterization of the DMC1 Gene in the Kufri Jyoti Potato Cultivar

**Figure 2:**
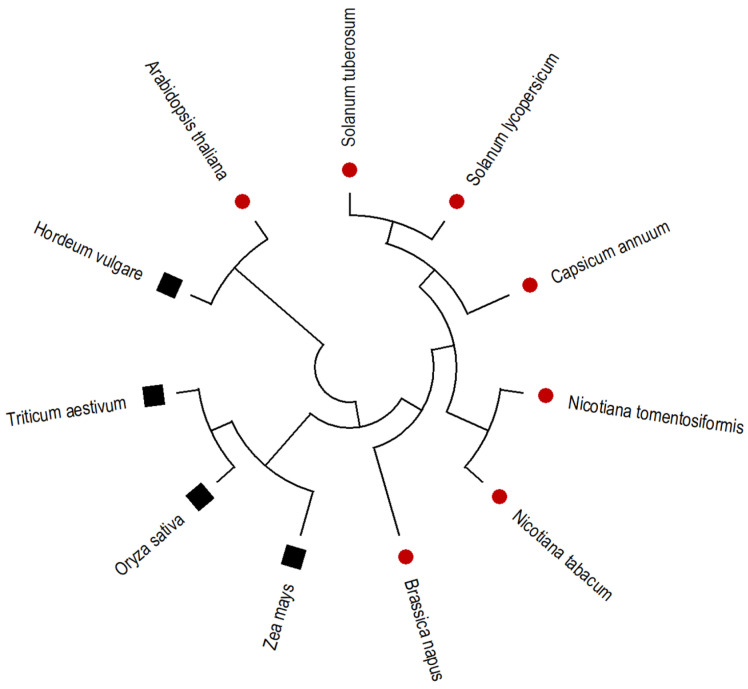
Phylogenetic analysis and comparison of the potato *StDMC1* gene sequence with that of major crops by the maximum likelihood method. In this phylogenetic tree, red color circles represent dicot species, and black squares represent monocot species.

The CDS sequence of the DMC1 gene from *Arabidopsis* was used as a query in the PGSC database (https://spuddb.uga.edu/index.shtml, accessed on 25 June 2021) via the BLAST tool. The CDS sequence of the *AtDMC1* gene showed 81% similarity with the diploid potato DMC1 CDS sequence. Primers designed from the gene sequence (Soltu.DM.09G025170.1) were used to amplify the DMC1 gene sequence in the tetraploid Kufri Jyoti cultivar. The sequence of Kufri Jyoti for the DMC1 gene was analyzed for sequence similarity using the NCBI nucleotide BLAST tool. The phylogenetic analysis carried out using MEGA 11.0.13 revealed that Kufri Jyoti’s DMC1 has high sequence similarity with *Solanum lycopersicum* and *Capsicum* spp., followed by *Nicotiana tomentosiformis* and *N. tabacum* (Figure 2).

### 3.2. Confirmation of pRI101 RNAi Construct

**Figure 3:**
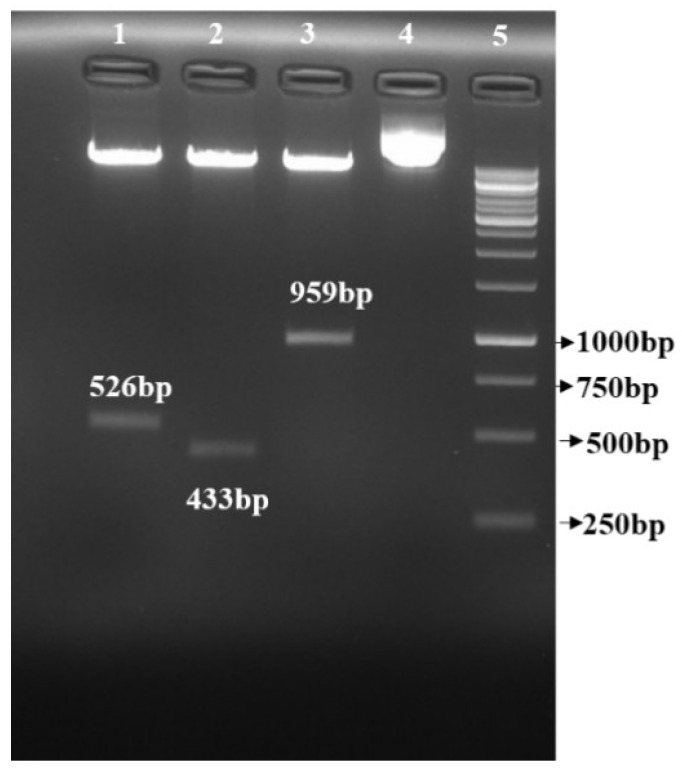
The plasmid containing the *StDMC1*:RNAi construct was digested with *Sal* 1 and *Kpn* 1 and *Bam* H1 and *Kpn* 1 restriction enzymes, respectively, to confirm sense and antisense insertion into the pRI101AN binary vector having the CaMV35S promoter and the NOS terminator. Lane 1: *StDMC1* sense with a product size of 526 bp digested with *Sal* 1 and *Kpn* 1, Lane 2: *StDMC1* antisense with a product size of 433 bp digested with *Bam* H1 and *Kpn* 1, Lane 3: Full cassette containing *StDMC1* sense::*StDMC1* antisense with a product size of 959 bp digested with *Sal* 1 and *Bam* H1, Lane 4: uncut pRI101AN plasmid with a product size of 10,417 bp, Lane 5: 1 Kb molecular marker.

The sense fragment was confirmed through colony PCR. A band of 1.3 Kb gene size (526 bp *StDMC1* sense::801 bp::35S CaMV::58 bp 5′UTR) confirmed the insertion of the gene in the vector (Figure S1). Likewise, the antisense fragment was confirmed using colony PCR. A band of 1.8 Kb fragment size (801 bp 35S CaMV::526 bp Sense *StDMC1*::58 bp 5′UTR::433 bp *StDMC1* antisense::264 bp NOST) confirmed the successful integration of the full gene cassette in the pRI101AN vector (Figure S2). The whole cassette (*StDMC1* sense::*StDMC1* antisense::pRI101AN) was reconfirmed through restriction digestion using *Sal* 1 and *Bam* H1 (Figure 3). The sense fragment, being complementary and longer than the antisense fragment, would turn back and form a hairpin loop for knocking down the gene function, i.e., gene silencing.

### 3.3. Agrobacterium-Mediated Plant Transformation

**Figure 4:**
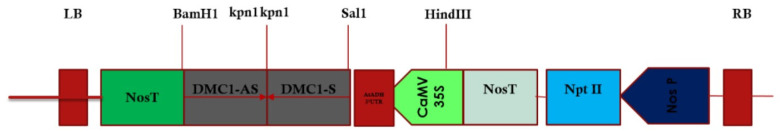
A schematic diagram of the RNAi vector construct. The hairpin structure consisting of a sense *StDMC1* sequence (oriented in *Sal* 1-*Kpn* 1 site) and the antisense *StDMC1* sequence (oriented in *Bam* HI-*Kpn* I site) was inserted between the (CaMV) 35S promoter and the nopaline synthase gene (NOS) terminator.

**Figure 5:**
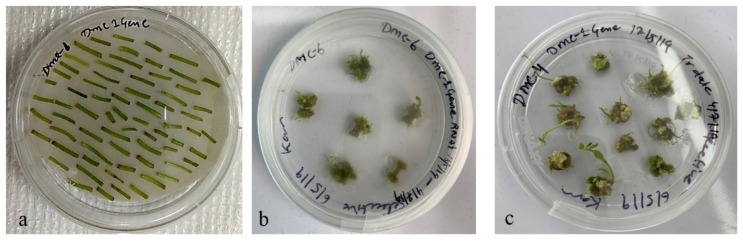
*Agrobacterium* harboring the *StDMC1* RNAi construct used for genetic transformation. (**a**) In vitro 15-day-old tissue culture internodal stem cuttings were placed on pre-selective basal MS medium for 48 h. (**b**) The treated cuttings were transferred to regeneration medium containing Indoleacetic acid (IAA) (1 mg/mL), Naphthalene acetic acid (NAA) (0.01 mg/L), Gibberellic acid (GA3) (3 mg/mL), Cefotaxime (100 mg/mL), Kanamycin (50 µg/mL), and Carbenicillin (100 mg/L) and plates were kept at 20 ± 2 °C under fluorescent light at 100 μmol m^−2^ s^−1^ with 16 h light and 8 h dark for callus formation. (**c**) Shoot development in putative transformants.

**Figure 6:**
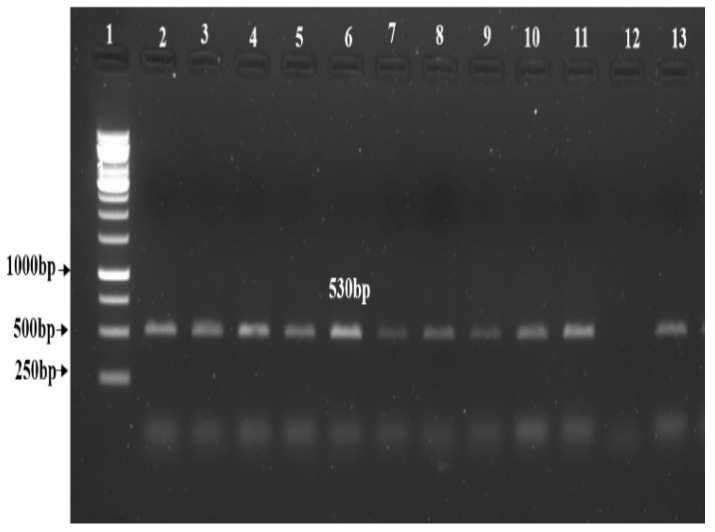
Validation of transformed lines by PCR amplification using NPTII primers resulting in a product size of 530 bp. Lane 1: 1 Kb Ladder, Lane 2–11: Transformed lines, Lane 12: Non-transformed line, i.e., Kufri Jyoti (control), and Lane 13: Positive plasmid containing *StDMC1* sense::*StDMC1* antisense:pRI101AN.

*Agrobacterium* strain GV3101 harboring the complete RNAi cassette carrying *StDMC1* sense::*StDMC1* antisense::pRI101AN (Figure 4) was used to transform the Kufri Jyoti internodal explants for the generation of DMC1 mutated lines. The callus formation was observed 40 days after the co-cultivation, followed by putative shoot generation (Figure 5). In total, 310 putative transgenic lines were obtained, which were grown in a glass house under controlled conditions and were screened using the NPTII marker. Out of 310 lines, 137 lines were found positive, with the NPTII marker showing a product size of 530 bp (Figure 6). Overall, a transformation efficiency of 44% was obtained for mutants (Table 3).

### 3.4. Pollen Viability

**Figure 7:**
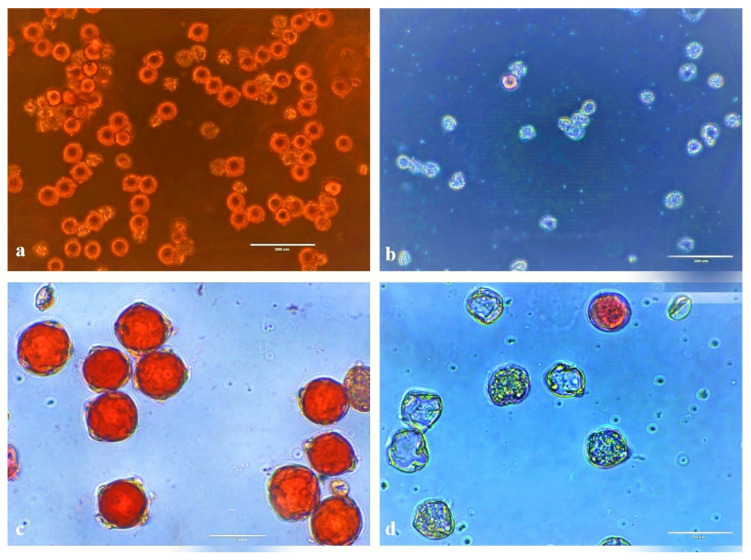
Pollen viability using acetocarmine (2%) under an EVOS XL Core Imaging System Microscope (Invitrogen^TM^). Five frames of each slide were examined to count the viable and non-viable pollen grains. Viable pollen grains retained the stain, whereas the non-viable pollen grains were unable to retain the stain. (**a**) Control “Kufri Jyoti” pollen grains (scale bar = 200 µm), (**b**) RNAi lines showing non-viable pollen (scale bar = 200 µm), (**c**) control “Kufri Jyoti” pollen grains (scale bar = 50 µm), and (**d**) RNAi lines showing non-viable pollen (scale bar = 50 µm).

The acetocarmine-based pollen staining revealed that four DMC1 mutant lines had reduced pollen viability of 14–21% in comparison to the Kufri Jyoti control plants, which showed 78% pollen viability. Out of 137 positive lines, four mutant lines showed reduced pollen viability (Table 4; Figure 7). All other lines exhibited moderate-to-high pollen viability, as observed in the control Kufri Jyoti plants. The results confirmed the generation of DMC1 knock-out mutant lines.

### 3.5. Quantitative Real-Time PCR (qRT-PCR)

**Figure 8:**
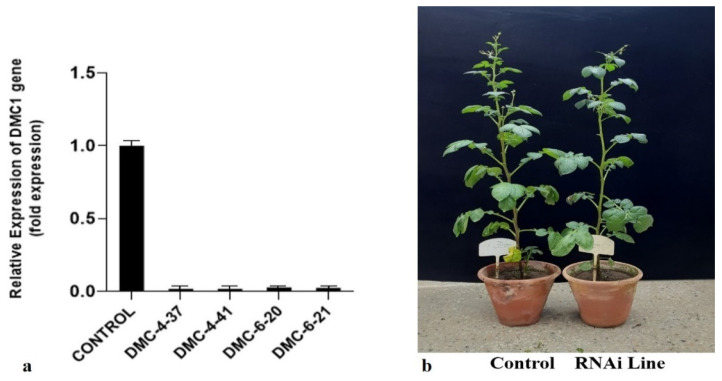
Real-time quantitative reverse transcription polymerase chain reaction (qRT-PCR) analysis of control vs. RNAi lines. The expression of the *StDMC1* gene in comparison to the average Ct values of the housekeeping gene (elf) was determined and analyzed using the 2^−ΔΔCt^ method. (**a**) Expression analysis of control vs. putative mutant lines. (**b**) The fully grown control (left) and RNAi lines (right) plants of Kufri Jyoti.

qRT-PCR was carried out to measure the expression level of the DMC1 gene in mutant lines as well as in the control plant. The results of qRT-PCR clearly showed that DMC1 gene expression was significantly downregulated in four putative RNAi mutant lines in comparison to the control plant (Figure 8a). The expression of the DMC1 gene was downregulated to levels of 60-fold in DMC4-37, 56-fold in DMC4-41, 35-fold in DMC6-20, and 42-fold in DMC6-21. These findings support the pollen viability data and indicate the silencing of the DMC1 gene in these Kufri Jyoti lines.

## 4. Discussion

TPS could be a popular choice for the commercial production of potatoes, since it is simple to handle, transport, and is virus-free; however, due to its genome complexity and autotetraploid nature, it cannot remain true to type through sexual reproduction. Therefore, the production of non-recombinant gametes by arresting the crossing over among non-sister chromatids could be the first step towards achieving parental-type gametes for reconstitution of true-to-type varieties through sexual reproduction in potatoes. Here we aimed to develop non-recombinant gametes of the potato cultivar Kufri Jyoti by silencing the expression of the *StDMC1* gene through RNAi hairpin loop technology. At first, we identified the DMC1 gene in potato through in silico analysis of *Arabidopsis thaliana AtDMC1* CDS, which showed 81% sequence identity with the *StDMC1* CDS sequence (Soltu.DM.09G025170.1). We found only one DMC1 gene on chromosome 9 in the potato genome (http://spuddb.uga.edu/index.shtml, accessed on 25 June 2021), similar to barley [30], wheat [31], and maize [27]. Phylogenetic analyses revealed that the *StDMC1* gene of *S*. *tuberosum* had more similarity with *Solanaceous* crops, viz., *S. lycopersicum* and *Capsicum annum*, followed by *Nicotiana tomentosiformis* and *N*. *tabacum*; however, the DMC1 gene from monocot species formed a separate cluster except for *Hordeum vulgare*. The DMC1 nucleotide sequences of *S. tuberosum, S*. *lycopersicum*, and *C. annum*, which are all members of the same family, were found to be the most identical. The results provide strong evidence that the isolated cDNA homologue employed in this study is an accurate orthologue of the potato DMC1 gene.

The RNAi technique has been successfully used in potatoes to knock down the function of various genes, including the beta-carotene hydroxylase gene [20,32], susceptibility factors for late blight [33], and RNA viruses [34]. However, meiotic cycle genes have not been targeted in any autopolyploid to date. Although DMC1 and its orthologues have been studied for their functions in *Arabidopsis* [35], rice [23], wheat [36], and barley [37], this is the first attempt in any autopolyploid crop, the potato, to silence the function of the meiotic cycle gene *StDMC1*. We also chose the same strategy to knock down the function of the important meiotic cell division gene *StDMC1* in the popular Indian potato variety Kufri Jyoti as followed earlier in rice [25]. Kufri Jyoti is an old but widely adapted popular potato cultivar in India and has been successfully used in transformation studies previously [38]. The RNAi cassette consisting of sense and antisense strands (*StDMC1* sense::*StDMC1* antisense::pRI101AN) was successfully transformed into the potato variety Kufri Jyoti via *Agrobacterium*-mediated transformation, as has been demonstrated in an earlier study [39]. We used Kanamycin (50 mg/L) in the selection medium for shoot formation, while Deng et al. [25] used Hygromycin B (40 mg/L) in *Oryza sativa.* The transformation efficiency after screening with NPTII primers was found to be 44% in Kufri Jyoti putative mutants, which was considerably higher in comparison to 10–16% in Kufri Chipsona 1 cultivar [40].

Although there were no morphological differences observed between the mutant lines and control plants (Figure 8b), the gametic cells (pollen grains) showed reduced viability in mutants in comparison to control plants. Four mutant lines showed pollen viability of 14–21%, indicating partial sterility. It is well known that the plants with a defective DMC1 gene show reduced pollen viability compared to the control plants [25]. A similar type of study in *Arabidopsis* has already proven that silencing the DMC1 gene can alter the shape of pollens and reduce their viability [41]. Barley plants carrying mutations in the *HvDMC1* gene lead to unusual synapsis and chromosomal missegregation [25,42]. Likewise, Tian et al. [43] performed cytological studies to decipher the function of *AtDMC1* as well as ASY1 in *Arabidopsis* and showed that polyploidy can potentially alleviate lethal effects brought by mutations during plant reproductive life cycles. This indicates that DMC1 mutants may revert to normal meiosis in potato, which have an autopolyploid genome.

The analysis of putative four mutants through quantitative real-time PCR (qRT-PCR) revealed substantial down-regulation of DMC1 gene expression in four mutant lines, DMC4-37, DMC4-41, DMC6-20, and DMC6-21, in comparison to control Kufri Jyoti plants, indicating that the RNAi construct was successful in silencing the DMC1 gene in these transformed lines. Similar down-regulation and low transcript levels for the DMC1 gene have been observed earlier in maize calli transformed by pART-DmRNAi [27].

**Figure 9:**
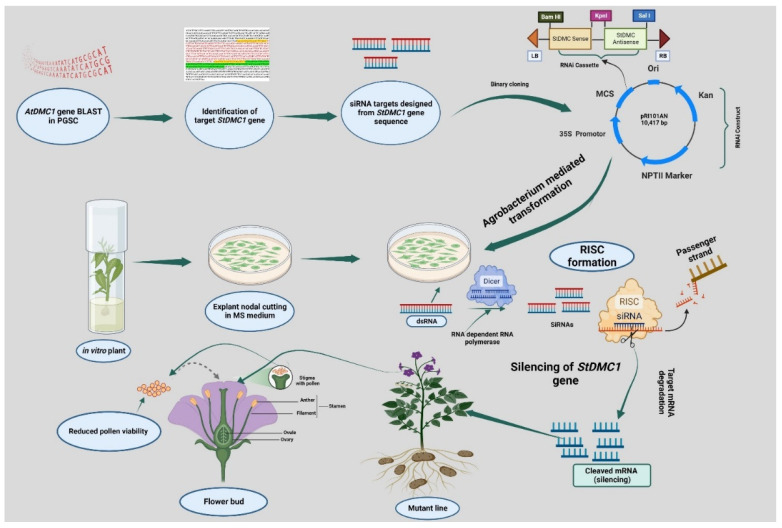
Schematic illustration of the mechanism of silencing of *StDMC1* gene through RNA interference (RNAi) technique. *Arabidopsis thaliana AtDMC1* CDS sequence was used as a query in the Potato Genome Sequencing Consortium (PGSC) database and the *StDMC1* gene sequence was retrieved for designing the siRNA target through in silico analysis. The target gene sequence was cloned in a binary vector, i.e., pRI101AN, and transferred in plants through *Agrobacterium*-mediated transformation. The process included shearing dsRNA into smaller dsRNA intermediates by the RNase-III type endonuclease known as Dicer. The small interfering RNA (siRNA) containing the complementary sequence of the target RNA acts as a guide RNA, and the other strand, known as the passenger RNA, gets degraded. The mutant lines were obtained by using internodal explant cutting on Kanamycin-selective MS medium. Reduced pollen viability was observed in silenced *StDMC1* gene lines.

The results revealed and verified the role of *StDMC1* in potatoes, which acts as a platform for the generation of non-recombinant gametes and could play an important role in the development of a TPS-based breeding program in potatoes. However, these lines need further characterization in advanced sexual generations for use as asynaptic mutants in potatoes. We have also summarized our results in Figure 9, which illustrates the experimental procedure for the silencing of the *StDMC1* gene in potatoes.

## 5. Conclusions

To prevent cross-over and recombination in potatoes, we used the RNAi method to target the production of *StDMC1* mutants. The CaMV35S promoter was employed to direct the creation of an RNAi construct using an efficient siRNA. The pollen viability of putative mutants was lower than that of the control, Kufri Jyoti plants. Four mutant lines showing partial pollen sterility also exhibited low expression levels of *StDMC1* in comparison to the control. These four lines will be further investigated in sexual generations for use as asynaptic mutants in potatoes. This is the first study on knocking down the function of the *StDMC1* gene in auto-tetraploid potato to develop asynaptic mutants. The characterization of four mutant lines generated in this study could open new research opportunities to study asynaptic mutants of potatoes. Our findings show that meiosis in *S*. *tuberosum* can be controlled to produce meiotic mutants and parental lines for TPS breeding. We also show how RNAi-mediated meiosis modification can pave the way for the creation of effective plant breeding techniques in potatoes.

## Figures and Tables

**Table 1:** Details of primers used in this study. The red color denotes the restriction sites of different enzymes in the primer sequences.

Primer	Sequence with Restriction Enzyme	Size (bp)	Enzymes
StDMC1 Sense-F	CGCGTCGACGAAGATAGTGAACTTCGGC	526	*Sal* 1
StDMC1 Sense-R	CCCGGGTACCCTTTATCAATCGGGACAGC		*Kpn* 1
StDMC1 Antisense-F	CGCGGATCCGAAGATAGTGAACTTCGGC	433	*Bam* H1
StDMC1 Antisense-R	CCCGGGTACCAGAATCCACAATCAGAAGTC		*Kpn* 1
NPT II-F	TTTGTCAAGACCGACCTGTC	530	
NPT II-R	CCAACGCTATGTCCTGATAG		

**Table 2:** Details of primers used in the real-time PCR (qRT-PCR).

Real-Time Primer	Sequence
StDMC RT-F	TACATTACTGGGGAGTGAGGC
StDMC RT-R	CCCCAAAAGCTTCAGTTATTGC
elf-F	CGTTGTATCATGAATTTGTTTCTCTGT
elf-R	CCCCCTGAGGTTTCAACG

**Table 3:** Transformation efficiency of potato cultivar “Kufri Jyoti” genetically transformed with *Agrobacterium* strain GV3101 harboring binary vector pRI101.

Explant (Internode)	No. of Cuttings	Callus	No. of Regeneration of Shoots	NPTII Positive	Percentage of Transformation Efficiency
Kufri Jyoti	140	100	310	137	44

**Table 4:** Pollen viability in putative DMC1 mutant lines.

Mutant Lines	Viable Pollens	Non-Viable Pollens	Total	% Viability
DMC4-37	18	107	125	14.4
DMC4-41	12	53	65	18.46
DMC6-20	26	97	123	21.14
DMC6-21	18	88	106	16.98
Kufri Jyoti- Control	112	31	143	78.32

## Data Availability

The author confirms that the data supporting the finding of this study are available within the article and its Supplementary Materials.

## Supplementary Materials

The following supporting information can be downloaded at: https://www.mdpi.com/article/10.3390/life13010174/s1. (Figure S1: Validation of *StDMC1*:sense fragment in pRI101AN binary vector using Colony PCR. The transformants were confirmed with 35S forward and gene specific reverse primer. The 1.3 Kb gene fragment (526 bp *StDMC1*:Sense + 801 bp 35S CaMV + 58 bp 5′UTR) confirmed the insertion of gene in vector and Figure S2: Validation of *StDMC1* sense::*StDMC1* antisense fragment in pRI101AN binary vector using Colony PCR. The transformants were confirmed with 35S forward and NOST reverse primers. The 1.8 Kb gene fragment (526 bp *StDMC1*: sense + 433 bp *StDMC1* antisense + 801 bp 35S CaMV + 58 bp 5′UTR) confirmed the insertion of gene in vector).
