# Supplementary figures and images for: Generation of Asynaptic Mutants in Potato by Disrupting StDMC1 Gene Using RNA Interference Approach
Source: Life (Basel). 2023 Jan 6;13(1):174. doi: 10.3390/life13010174 (PMC9861435; doi:10.3390/life13010174)

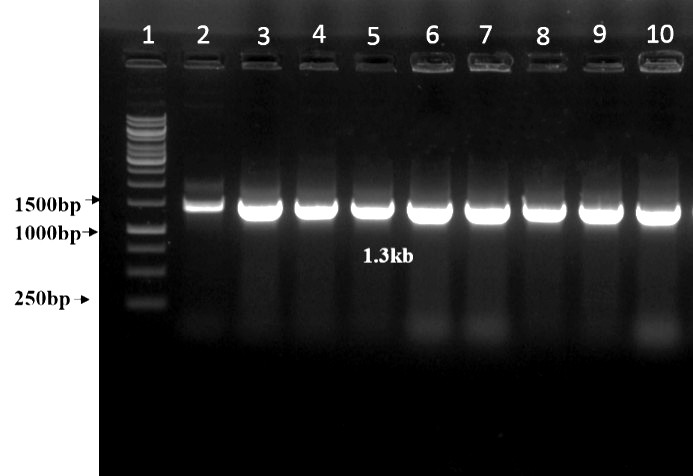

Supplement: Supplementary file 1 [file life-13-00174-s001.zip › life-2034968-supplementary/Fig-S1.jpg]

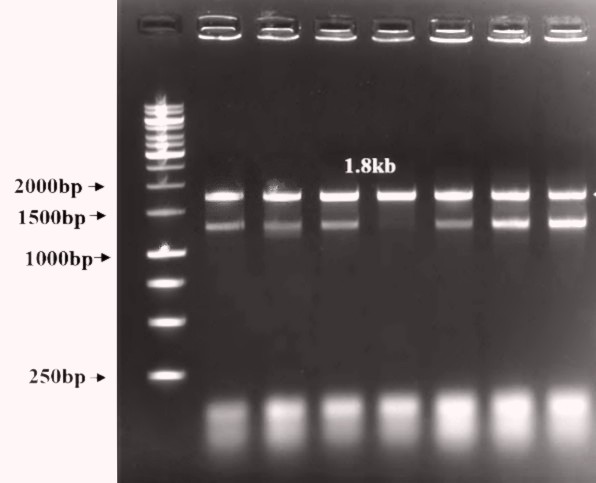

Supplement: Supplementary file 1 [file life-13-00174-s001.zip › life-2034968-supplementary/Fig-S2.jpg]
